# Indigenous community-based approaches to environmental justice through citizen science

**DOI:** 10.1007/s00550-026-00588-2

**Published:** 2026-03-02

**Authors:** Afnan Agramont, Analy Baltodano Martinez, Mohammad Gharesifard, Leonardo Villafuerte Philippsborn, Liliana Lizarazo-Rodriguez, Stuart Warner, Ann van Griensven

**Affiliations:** 1 Water and Climate Department, Vrije Universiteit Brussels, Brussels, 1050 Belgium; 2 Institute on Comparative Regional Integration Studies, The United Nations University(UNU-CRIS), Bruges, 8000 Belgium; 3 Faculty of Science and Engineering, University of Groningen, Nijenborgh 7, Groningen, 9747 AG The Netherlands; 4 Instituto para la Democracia (IpD) de la Facultad de Derecho y Ciencias Políticas, Universidad Católica Boliviana San Pablo, Av. 14 de Septiembre y calle 2 de Obrajes, La Paz, Bolivia; 5 Law and Development Research Group, University of Antwerp, Antwerp, 2000 Belgium; 6 Brussels School of Governance, Vrije Universiteit Brussel, Brussels, 1050 Belgium; 7 Global Environment Monitoring Unit, United Nations Environment Programme (UNEP), Nairobi, Kenya; 8 Department of Water Science Engineering, IHE-Delft Institute for Water Education, Delft, 2611 AX The Netherlands

## Abstract

The Katari River Basin, a key watershed feeding Lake Titicaca, is severely contaminated due to mining waste, urban effluents, industrial discharges, and agricultural runoff. These pressures have disproportionately affected downstream Indigenous Aymara communities, threatening their rights to clean water, food security, and cultural continuity. This study examines citizen science as a participatory approach through which Indigenous communities engage with environmental justice concerns in their territories. Through a participatory process involving local community members, 46 water samples were collected over a four-month period and analysed using low-cost monitoring methods. Results show consistent exceedances of Bolivia’s national water quality standards, particularly for total dissolved solids, phosphate, and turbidity, with the most severe breaches occurring in the Katari River. Findings were presented and discussed during community workshops on Indigenous and environmental rights, facilitating collective interpretation and dialogue. These workshops were complemented by 20 semi-structured interviews exploring how participants responded to the scientific evidence in relation to their lived experiences. A thematic analysis reveals that citizen science can foster environmental knowledge, intergenerational dialogue, and awareness of environmental rights and responsibilities, while remaining insufficient on its own to generate distributive or institutional environmental justice outcomes in the absence of legal literacy, institutional responsiveness, and formal accountability mechanisms. Drawing on a resources nexus lens, the findings also highlight the interdependence of water quality, food systems, and cultural integrity, emphasising the need for rights-based, community-anchored approaches to environmental governance in Indigenous contexts.

## Introduction

 Water pollution represents a significant threat to both ecological integrity and human health, particularly in regions facing rapid urban and industrial expansion, inadequate infrastructure, and governance challenges (Nakkazi et al. 2024; van Vliet et al. 2023). Understanding these challenges through a resources nexus perspective, which examines the interconnectedness and interdependencies among systems such as water, energy, food, and ecosystems, helps comprehend how impacts in one resource, such as water, can cascade through others (Kurian 2017; Liu et al. 2018) This perspective is especially pertinent in contexts marked by environmental injustice, where the uneven distribution of impacts intersects with political marginalization.

In the Andean region of Bolivia, the Katari River Basin is experiencing severe contamination from upstream mining waste, urban wastewater, industrial discharges, and agricultural runoff (Agramont et al. 2019; Baltodano et al. 2022). These environmental pressures have degraded water quality and reduced river flows feeding into Lake Titicaca, contributing to the lake’s ongoing ecological decline and threatening the most important freshwater resource in the Andean region (Agramont et al. 2022). The impacts of pollution are asymmetrically distributed; downstream Indigenous Aymara communities are disproportionately exposed to environmental hazards that threaten access to clean water, disrupt livelihoods and food security, and compromise essential local ecosystem services (Bullard 2001; McGregor et al. 2020; Ridzuan 2021). This systemic inequity stresses the need for inclusive and participatory rights-based approaches to environmental monitoring (Boelens et al. 2023; Norman 2018).

Although Bolivia’s constitution and several international agreements formally recognize Indigenous environmental rights, their practical implementation remains limited (Parsons et al. 2021; Schilling-Vacaflor 2019b; Suman 2021). In practice, environmental regulations are unevenly enforced, particularly in upstream urban and industrial areas, and affected Indigenous communities often lack timely access to official water quality data generated by state agencies. As a result, communities face structural barriers to demonstrating harm, engaging regulatory authorities, and demanding accountability, creating a gap between the formal recognition of rights and their realization on the ground.

Citizen science, defined as the active, often collaborative involvement of non-professional scientists in research processes ranging from problem identification to data analysis and dissemination, has emerged as a promising tool to democratize environmental knowledge and enhance local governance (Bonney et al. 2009; Vohland et al. 2021). Yet, its potential to support Indigenous environmental justice and contribute to understanding resource interdependencies remains underexplored (Fernández-Llamazares et al. 2020; Gharesifard et al. 2025; King et al. 2021). In many contexts, citizen science is still perceived primarily as a data collection mechanism rather than as a platform for producing actionable evidence to support rights claims or redress environmental harms (Dosemagen & Parker, 2019; Gharesifard et al. 2019).

In this article, environmental justice is not treated as a singular outcome, but as a multidimensional analytical concept. The study distinguishes between environmental injustice as a structural condition experienced by Indigenous communities, environmental justice as a set of procedural and recognitional processes enabled through participation, and environmental justice as a normative horizon that remains contingent on broader institutional change.

This study addresses that gap by exploring the potential of community-based water quality monitoring to promote environmental justice in the Katari River Basin. Through a participatory process involving local Indigenous participants, the project pursued three objectives: (a) to determine and monitor key water quality parameters at the community level, (b) to build Indigenous youth capacity in low-cost and accessible water quality testing methods, and (c) to interpret findings against the benchmarks set by national water quality standards and environmental rights frameworks. These objectives guided the following research questions: (1) What are the current water quality conditions in the Katari River Basin, as measured through citizen science? (2) How participants interpret and respond to these findings in relation to Indigenous rights and environmental justice?

Framed through a nexus perspective, this research explores how participatory water monitoring enables participants to interpret interconnections among the Sustainable Development Goals (SDGs), including clean water and sanitation (SDG 6), reduced inequalities (SDG 10), climate action (SDG 13), life below water (SDG 14), life on land (SDG 15), and strong institutions for justice (SDG 16) (Fritz et al. 2019; Gharesifard et al. 2025). In this study, the resources nexus is employed primarily as an interpretive lens rather than as a prescriptive analytical framework guiding data collection. Nexus interdependencies are examined inductively through participants’ narratives and reflections on lived experience, rather than through predefined systems categories.

The remainder of the paper is structured as follows. Section 2 reviews the literature on water pollution asymmetries, Indigenous environmental justice, and citizen science, establishing the conceptual framework for the study. Section 3 presents the study area and methodology, detailing the community-based water quality monitoring and qualitative data collection. Section 4 reports the water quality results and insights from participant interviews. Section 5 discusses the findings through an Indigenous environmental justice and resource nexus lens, and Sect. 6 concludes with implications for participatory monitoring and environmental governance.

## Literature review

This section reviews the literature that conceptually anchors the study, moving from structural water pollution asymmetries, to Indigenous environmental justice and rights, and finally to citizen science as a socio-technical approach situated at the intersection of these debates.

### Water pollution asymmetries at the centre of a nexus challenge

Water pollution is a growing global crisis with severe implications for both human health and the environment. The complex and escalating interplay of global change factors, including climate change, population growth, urbanization, and industrialization, is exacerbating the severity of this crisis. The contamination of water bodies by pollutants, such as industrial waste, agricultural runoff, and municipal sewage, results in a range of adverse nexus challenges. These include the proliferation of waterborne diseases, the disruption of ecosystems, and the depletion of vital aquatic resources. Various studies point to this dangerous situation of freshwater change worldwide, noting that planetary boundaries are being exceeded due to human impacts on the freshwater cycle (Nakkazi et al. 2024; Nkwasa et al. 2024; van Vliet et al. 2023).

Improving water quality relies heavily on monitoring processes that provide a better understanding of pollution dynamics, source-impact relationships (Baltodano et al. 2022), and the need for infrastructure and regulations (Hannah et al. 2022). However, national capacities for water quality monitoring are highly asymmetrical worldwide. This disparity is evident in submissions for Sustainable Development Goal (SDG) indicator 6.3.2 on ambient water quality, in which the poorest half of the world contributed less than 3% of the total water quality measurements (UNEP, 2024).

Moreover, water contamination disproportionately affects vulnerable and marginalized communities, often characterized by low income and racial or ethnic minorities (Bullard 2001; Liddie et al. 2023). This deepens existing inequalities, making these communities more susceptible to health risks and economic hardships (Hoque et al. 2021; Jorgenson 2004; Ridzuan 2021). The asymmetrical distribution of water pollution is a resources nexus challenge that reflects not only economic disparities (SDG 10) but also exposes affected communities to health problems (SDG3), climate-driven vulnerabilities (SDG 13), and biodiversity loss (SDGs 14 and 15). Indigenous populations in high-altitude basins like Katari are particularly at risk due to glacial melt, shifting precipitation patterns, and the increasing frequency of extreme weather events, all of which compound pre-existing pollution stressors(Adler et al. 2023). Addressing these inequalities requires a system thinking approach and institutional governance mechanisms that integrate local monitoring efforts into national policy frameworks, ensuring fair access to environmental protection (SDG 16).

The inequitable distribution of environmental benefits, hazards and harms is a central concern of environmental justice (Hornborg & Martinez-Alier, 2016). The main concern is to ensure that everyone, regardless of their socioeconomic status or background, has the right to a healthy and safe environment (Boelens et al. 2023; Newell 2005), and is not exposed to environmental hazards in a disproportionate manner. Water contamination severely threatens crucial ecosystem services, including nutrient cycling, water purification, and habitat provision. This disruption destabilizes ecosystems, triggers biodiversity loss, and reduces their resilience to environmental stressors, resulting in serious consequences for both humans and nature.

These asymmetries are not merely technical failures but raise fundamental questions of environmental justice, particularly for Indigenous communities whose livelihoods and cultural integrity depend on affected water systems.

### Indigenous water rights and injustices

Indigenous groups are frequently confronted with situations of vulnerability and marginalization due to a confluence of historical and contemporary factors (Parsons et al. 2021). Many have experienced historical and systemic discrimination, including land dispossession, forced assimilation, and barriers to accessing natural resources, education, healthcare, and economic opportunities (Axelsson & Sköld, 2006). These realities stress the necessity of inclusive governance mechanisms that institutionalize grassroots participation in decision-making processes (Suman 2021). Additionally, many Indigenous communities live in regions that are highly vulnerable to climate change and other environmental pressures. This vulnerability is not a result of poor land stewardship but reflects global climate dynamics that affect even well-preserved ecosystems. Moreover, industrial activities and inadequate waste management near Indigenous territories often exacerbate local environmental pressures, increasing their exposure to rights violations (Abate & Kronk, 2013; Hornborg & Martinez-Alier, 2016) and highlighting the urgency of improving their access to environmental justice.

Indigenous communities tend to be deeply connected to their lands and waters (Norman 2018; Parsons et al. 2021). Pollution generated by activities such as large urbanization, industrial waste, agricultural runoff, or mining operations, disproportionately affects Indigenous and local communities (McGregor et al. 2020; Schlosberg et al., 2010; Tsosie 2007). They are frequently confronted with limited access to safe drinking water, leading to a higher incidence of waterborne diseases like diarrhoea, typhoid, and cholera (Ortiz-Prado et al., 2022). In addition, water pollution also affects other aquatic resources, disrupt traditional food sources and livelihoods (Rivera Gironas et al. 2024) that also include socio-cultural and spiritual spheres of these communities (Fernández-Llamazares et al. 2020; Lizarazo-Rodriguez et al. 2024; Viaene 2021). This intersection of water pollution asymmetries with broader resource interdependencies emphasizes the relevance of a resources nexus perspective.

Indigenous communities often find themselves disadvantaged in their efforts to address water pollution considering river basin hydrological spatial and temporal dynamics (Agramont et al. 2022). They also frequently face systemic barriers that prevent effective participation in decision-making, access to justice, or contribution to environmental monitoring data considered by appointed institutions (Suman et al. 2023). Indigenous communities may also encounter significant obstacles in advocating for their rights and holding polluters accountable because of limited resources, lack of awareness of the legal remedies they can trigger (Emanuel & Wilkins, 2020), or because they are reluctant to use existing legal mechanisms to claim this protection (Pinto 2021).

This reality contrasts with international recognition of the human right to drinking water and sanitation as essential for the full enjoyment of life and human rights (UN General Assembly resolution 64/292 of 2010). In the context of the interaction between water and biodiversity, the Kunming-Montreal Global Biodiversity Framework (CBD/COP/DEC/15/4 of 2022) recognizes the importance of water for biodiversity and the crucial role of Indigenous and local communities in its conservation. Interactions between Indigenous communities, their territories and freshwater have also been recognized and protected by international legal standards. Various international instruments, both binding and non-binding, such as the International Labour Organization Convention 169 (1989) on Indigenous and Tribal Peoples (ILO 169), the American Declaration on the Rights of Indigenous Peoples 2016) (OASDRIP), and the United Nations Declaration on the Rights of Indigenous Peoples (2007) (UNDRIP), reinforce the rights of Indigenous peoples to consultation—and, in some cases, to free prior and informed consent, particularly when projects or state action may affect their territories, including watercourses. However, enforcement is often weak, and the anticipated benefits have not always materialized for these communities (Flemmer & Schilling-Vacaflor, 2016; Schilling-Vacaflor 2019b). Indigenous rights to their lands, as well as the obligations to consult them or obtain their consent, form part of the concept of procedural environmental justice (Bell & Carrick, 2017). This procedural dimension complements the substantive component, which, in legal terms, affirms that every human being has the right to a healthy life, a right intrinsically linked to a healthy environment. In Latin America, this right has been recognized in several key instruments, including the San Salvador Protocol to the American Convention on Human Rights, the Escazú Agreement on Access to Information, Public Participation and Justice in Environmental Matters in Latin America and the Caribbean, and enshrined in nearly all national constitutions in the region.

The systematic pollution of the Katari River Basin in Bolivia, which is the case study of this article, unveils the difficulties of granting access to environmental justice, and the impact of institutional complexities. Bolivia stands out as one of the few countries worldwide whose constitution recognizes a plurinational state, reflecting the fact that a significant proportion of its population belongs to Indigenous communities. The constitution also recognizes the fundamental right to water, which is essential for all living species, not just humans, and stipulates environmental protection as a means to safeguard the rights of present and future generations. However, these regulatory frameworks do not necessarily translate into effective respect for these rights and principles (Dolhare & Rojas-Lizana, 2023; Pinto 2021). Many communities remain unaware of these constitutional rights and international standards recognizing the importance of protecting water sources and their intrinsic relationship with Indigenous and other local communities. The state’s limited capacity to protect Indigenous rights has further exacerbated these communities’ vulnerability to the harmful effects of pollution (McGregor et al. 2020). These persistent gaps between formal recognition of Indigenous water rights and lived realities create the conditions in which alternative knowledge-producing approaches, such as citizen science, are increasingly mobilised.

### Intersecting citizen science and Indigenous environmental advocacy

Citizen science has emerged as a participatory approach that democratizes knowledge about socio-ecological systems, challenging dominant models of science and expertise. Traditionally, water quality assessments have relied on specialized skills, sophisticated laboratory instrumentation, and advanced physicochemical analyses. As a result, environmental data collection has remained the domain of governmental agencies, formal research institutions, and laboratories. However, advances in participatory methodologies and the development of cost-effective water monitoring technologies have expanded the feasibility of conducting water quality assessments beyond conventional approaches. These innovations have positioned citizen science as a valuable complement to conventional monitoring strategies, particularly in regions where state-led data collection efforts remain insufficient or absent. The United Nations Environment Programme (UNEP) has recognized the role of citizen science in improving spatial and temporal coverage of ambient water quality monitoring (SDG 6.3.2) while fostering broader stakeholder engagement in water governance (SDG 16) (Fritz et al. 2019). Furthermore, integrating local ecological knowledge with participatory data collection strengthens community resilience against climate-driven water stress (SDG 13).

Collaborations between professional scientists and local communities through participatory science strengthens environmental knowledge co-production and facilitate the inclusion of non-expert actors in scientific inquiry (Vohland et al. 2021). Citizen science helps reduce data gaps (Hegarty et al. 2021), improves measurement accuracy through participatory verification mechanisms (Capdevila et al. 2020; Quinlivan et al. 2020)​, and incorporates diverse analytical methodologies into water quality assessments (Ramírez et al. 2023). Additionally, citizen science strengthens partnerships between scientific institutions and local communities (Dosemagen & Parker, 2019), facilitates environmental scientific literacy (Branchini et al. 2015), and contributes to equity and inclusion within environmental governance (King et al. 2021). Despite these well-documented benefits, its potential to advance environmental justice among Indigenous groups remains largely unexplored.

Indigenous rights, particularly in relation to the environmental governance, involve the equitable distribution of environmental benefits and burdens, the formal recognition of Indigenous knowledge in decision-making processes, and the assurance of procedural justice in environmental policymaking (Agyeman et al. 2016; Bell & Carrick, 2017; Schlosberg 2013; Schlosberg & Carruthers, 2010). In many cases, Indigenous communities experience systemic environmental injustice, bearing a disproportionate share of the adverse impacts of extractive industries, industrial pollution, and weak regulatory and enforcement mechanisms to address these hazards (Etchart 2022; Flemmer & Schilling-Vacaflor, 2016; Schilling-Vacaflor 2019a). Besides the disproportionate burden of environmental degradation, the access to institutional mechanisms for redress and remediation is limited or not necessarily effective (Cambou & Buhmann, 2025; Fernández-Llamazares et al. 2020; Kennedy et al. 2023).

Citizen science offers Indigenous communities a means to document environmental degradation, generate empirical evidence, and advocate their rights within legal and policy frameworks. This potential is particularly evident when Indigenous groups engage in citizen science-based water quality monitoring that produces scientifically credible data. Community-based research initiatives can enable the integration of local ecological knowledge with scientific methodologies, fostering co-produced knowledge that reflects localized environmental realities and socio-cultural contexts (Gharesifard et al. 2019)​. Such participatory approaches strengthen community agency by identifying synergies and trade-offs across interlinked resources such as water, food, energy and environment, while also facilitating empirical data collection on water contamination to support advocacy for more integrated and sustainable natural resources management (Gharesifard et al. 2025)​. The active participation of Indigenous communities in data generation may enhance their capacity to contest environmental injustices and articulate claims for regulatory intervention, but such outcomes remain contingent upon legal literacy, institutional uptake, and the existence of enforceable accountability mechanisms.

The literature on participatory governance highlights the significance of integrating citizen-generated data into policy and decision-making processes (Wehn et al. 2021a)​. Despite the direct implications of water policies for Indigenous livelihoods, conventional water governance structures frequently exclude Indigenous voices, despite the direct implications of water policies for Indigenous livelihoods (Agramont Akiyama et al. 2022a). The inclusion of community-generated environmental data within formal governance frameworks constitutes a means of addressing this exclusion (Suman 2021), reinforcing the legitimacy of Indigenous claims, and ensuring that their perspectives shape environmental policymaking. Empirical studies have demonstrated that citizen science enhances the visibility of marginalized communities in governance structures, facilitates knowledge transfer between local actors and policymakers, and strengthens institutional accountability in environmental management (Wehn et al. 2021b)​. In legal contexts, community-led environmental monitoring has provided critical evidence in litigation against industrial polluters and in negotiations for environmental remediation measures (Rea et al. 2024).

The role of citizen science in supporting Indigenous rights extends beyond data collection. In some cases, local communities have employed participatory monitoring initiatives to contest environmental injustices resulting from industrial waste discharge, mining activities, and agricultural pollution (Gardner-Frolick et al. 2022; Temper et al. 2018). Furthermore, citizen science has been employed in negotiations with governmental agencies to ensure compliance with environmental protection laws and international human rights frameworks (Reyes-García et al. 2022). In this context, citizen science functions as a socio-technical approach that can support Indigenous advocacy efforts by producing situated knowledge and evidentiary resources, while not constituting a political instrument capable of advancing sovereignty or justice outcomes on its own.

Nonetheless, several challenges persist in implementing citizen science initiatives aimed at supporting Indigenous rights. Issues related to data sovereignty, the institutional legitimacy of community-generated data, and the sustainability of monitoring programs beyond short-term funding cycles require careful consideration (Wehn et al. 2021b). Additionally, the continued dominance of western scientific paradigms in environmental governance poses risks of epistemic exclusion, where Indigenous knowledge systems are devalued or overlooked (Gharesifard et al. 2019)​. Co-designing methodologies that integrate Indigenous worldviews with scientific approaches is a critical strategy for ensuring the ethical and effective implementation of citizen science initiatives within Indigenous territories.

To analytically structure these debates, this study adopts a multidimensional environmental justice framework drawing on David Schlosberg. Within this framework, environmental justice comprises three interrelated dimensions: distributive justice (the allocation of environmental harms and benefits), procedural justice (access to participation and decision-making), and recognitional justice (acknowledgement of Indigenous identities, knowledge systems, and lived experience) (Schlosberg 2004). This framework is used not to assume justice outcomes, but to distinguish which dimensions are empirically examined in this study and which remain structurally constrained.

Specifically, distributive and intergenerational injustices are examined through water quality data and participants’ narratives of long-term environmental degradation, while procedural and recognitional dimensions are explored through engagement in citizen science and the interpretation of monitoring results. Institutional accountability and remediation are treated as aspirational justice dimensions lying beyond the immediate scope of the initiative.

## Methodology

The study adopted a participatory mixed-methods research design, in which a community-based citizen science initiative functioned as the primary empirical setting for data generation. The project was designed as a research process that combined (i) community-led water quality monitoring, (ii) participatory workshops, and (iii) qualitative interviews to examine how Indigenous participants interpreted scientific evidence in relation to environmental justice and Indigenous rights. The quantitative component generated empirical evidence on water quality conditions (RQ1), while qualitative methods, including participatory workshops and semi-structured interviews, were used to analyse how participants interpreted and responded to this evidence in relation to Indigenous rights and environmental justice (RQ2).

Data collection took place between February and August 2024 in the Indigenous subcentral of Chojasivi, Katari River Basin, Bolivia, and involved collaboration between community members, local educators, and researchers from the Vrije Universiteit Brussel and the Bolivian Catholic University. A purposive sampling strategy was used to select participants directly involved in the citizen science process and community decision-making. High school students were selected in consultation with community authorities due to their availability, interest, and the community’s emphasis on intergenerational knowledge transfer. Community authorities and adult community members were included to capture governance and advocacy perspectives linked to water pollution impacts.

### Study area

**Fig. 1:**
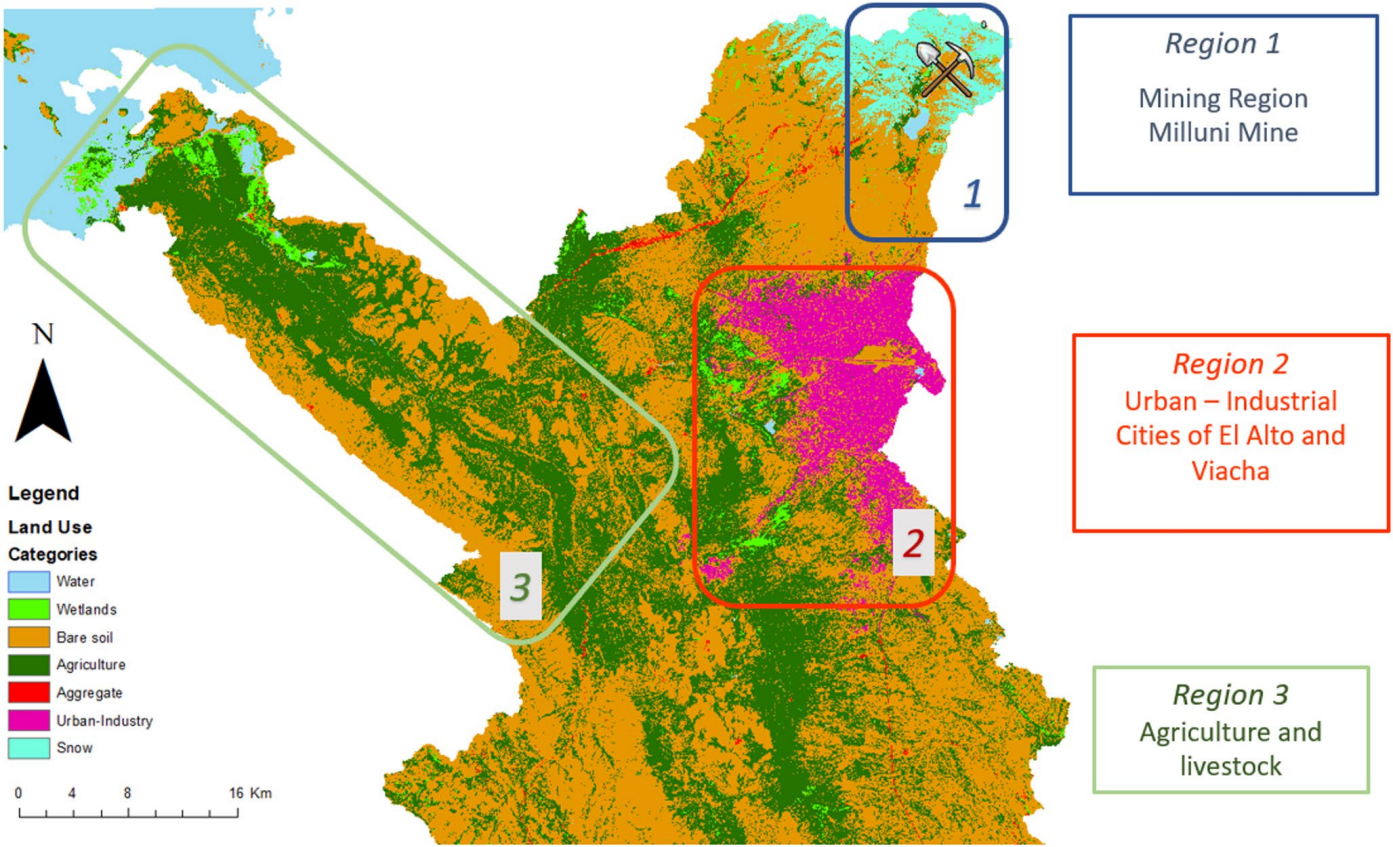
Spatial distribution of major anthropogenic environmental pressures in the Katari River Basin

The Katari River Basin is located in La Paz, Bolivia, and discharges into Lake Titicaca, the most important water resource in the Andes region. At the highest part of the river basin, over 100 years of mining operations have left a legacy of pollution (Archundia et al. 2017), see Fig. 1. Approximately two million tons of mining waste are constantly generating acid mine drainage (Agramont et al. 2019), significantly impacting the water quality of the entire system and introducing ecotoxicological contaminants into the downstream ecosystem’s food web (Gloria Rodrigo et al. 2018).

In the middle section of the river basin, the city of El Alto is the second most populated city in Bolivia and one of the fastest-growing cities in the world (Baltodano et al. 2022). This rapid growth has challenged the local administration to develop adequate sanitation services. While significant efforts have been made to improve water and sanitation services, El Alto currently has only 55% sewer coverage (Archundia et al. 2016). As a result, an estimated twenty million cubic meters of urban wastewater are discharged into the rivers flowing through the city (Agramont et al. 2022). Additionally, the city’s large industrial sector contributes liquid and solid waste to the river basin, given that it hosts most of the region’s industries. Moreover, the solid waste collection system has a coverage of approximately 63%, leading to the annual discharge of around eight million tons of solid waste into the rivers crossing El Alto (Agramont Akiyama et al. 2022b).

All of this aggregated pollution is carried by the rivers and eventually discharges into Lake Titicaca (See Fig. 1), impacting many indigenous Aymaran communities, including the Subcentral Chojasivi (Rivera Gironas et al. 2024).

### Participatory research design and data collection

The water quality data was collected in a period of four months, from March to June 2024. A total of 20 semi-structured interviews were conducted across three rounds (April 25, May 29, and August 28, 2024). Interviews involved high school students participating in water quality monitoring (*n* = 12), teachers supporting the activities (*n* = 2), and community members and authorities engaged in workshops (*n* = 6). Interviews lasted approximately 30–60 min and followed a semi-structured guide designed to ensure comparability while allowing participants to articulate their experiences and interpretations. The study applied a mixed-methods approach (Halcomb EJ & Hickman L, 2015) to assess not only water quality trends (SDG 6) but also their implications for health (SDG 3), social inequality (SDG 10), climate vulnerabilities (SDG 13), and governance challenges (SDG 16). Interview questions focused on four core themes: (1) perceptions of water quality and pollution, (2) experiences and learning through participation in citizen science, (3) awareness of environmental justice and responsibility, and (4) perceived community impacts and possibilities for advocacy.

**Fig. 2:**
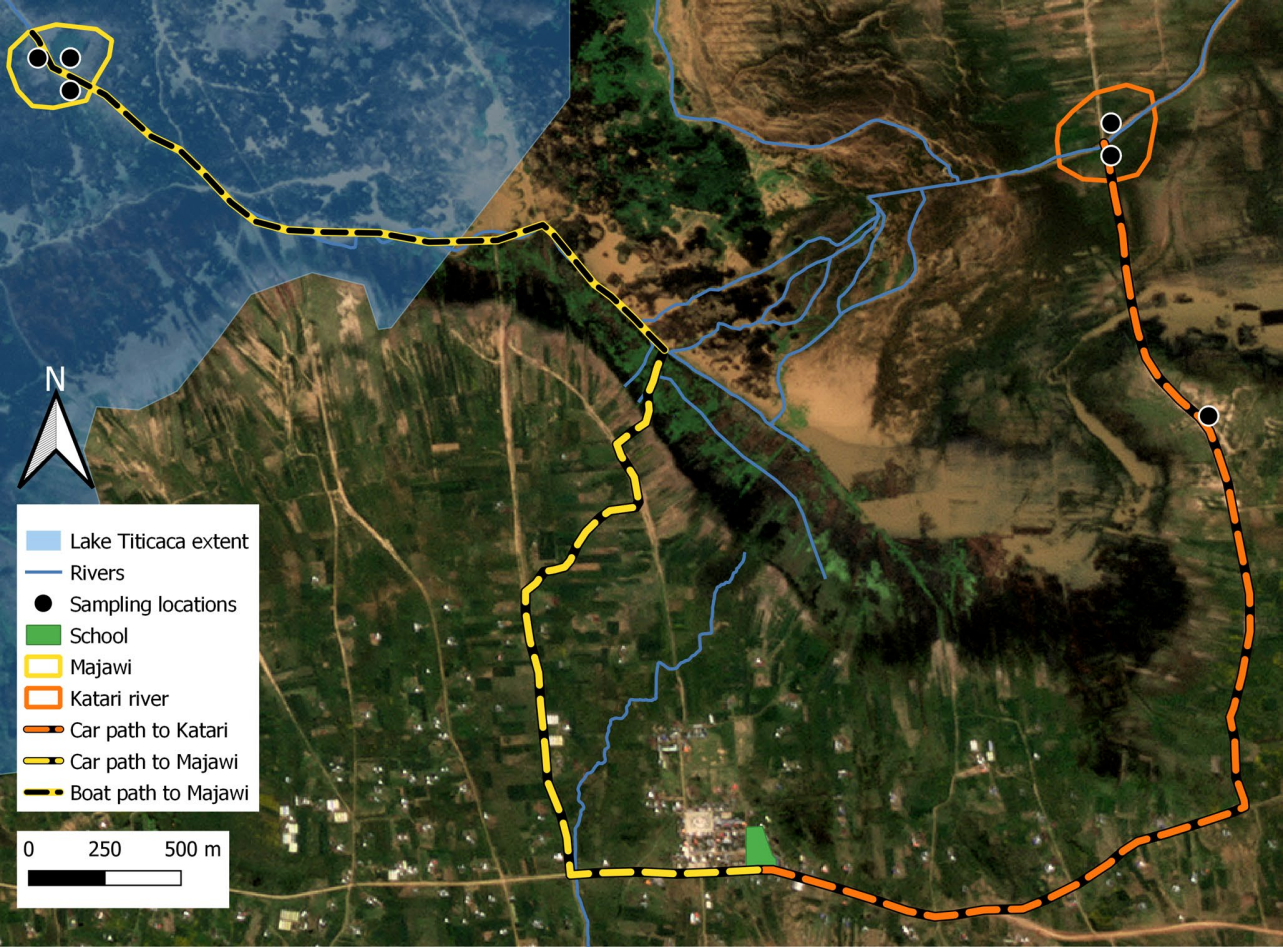
Water quality sampling sites and access paths from the school to the monitoring points

**Fig. 3:**
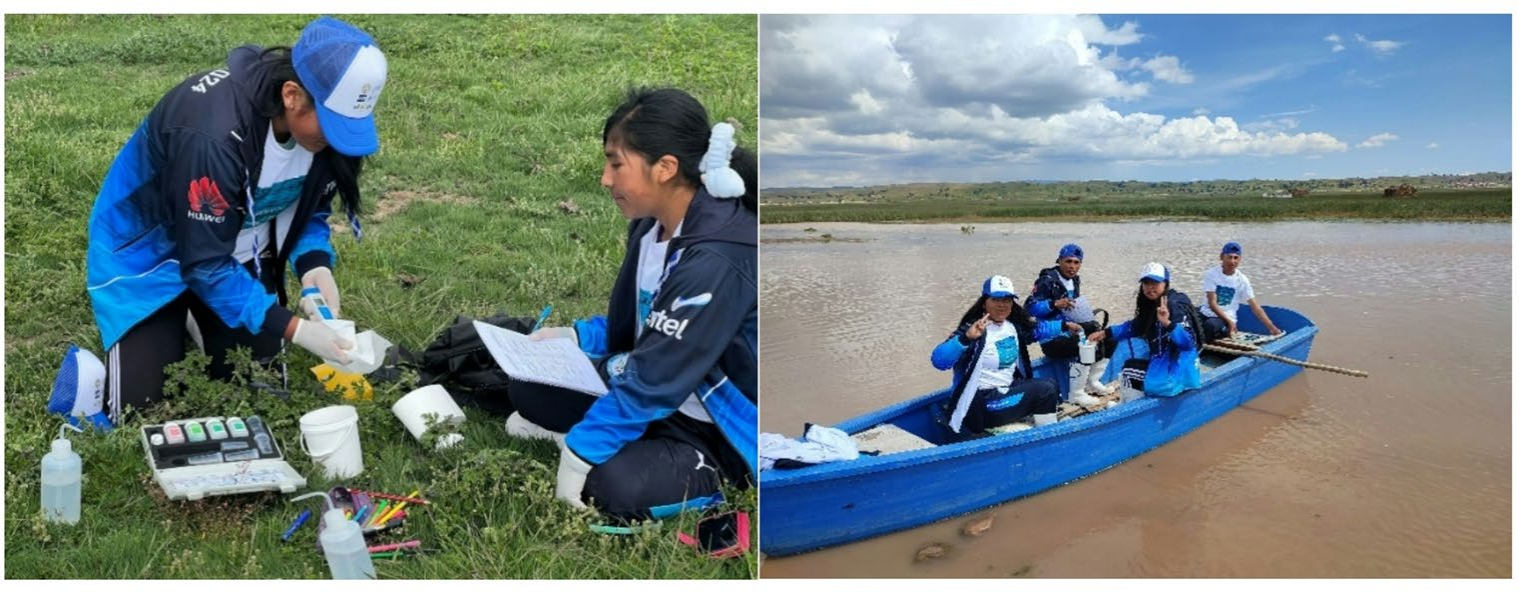
Community members performing water quality monitoring

The research project began in February 2024 with a participatory workshop that brought together eleven participants, three community authorities, the local high school principal, three community members, and four researchers, two from the Bolivian Catholic University and two from the Vrije Universiteit Brussel. This workshop focused on co-designing the citizen science research framework. The outcomes of the meeting included the following agreements: (1) high school students were chosen as primary participants in the study, due to their availability and the community’s interest in equipping the next generation with scientific skills; (2) two water quality monitoring sites were identified: one upstream at the community bridge and the other downstream near the grazing areas (See Fig. 2); (3) one water quality measurement per month to avoid the disruption of the school program, and (4) all the results obtained and their analysis were to be presented to the community in a public event.

A total of nineteen high school students from Unidad Educativa Técnico Humanístico “Elizardo Pérez” participated in the monitoring campaign, out of which seven were female (37%) and twelve were male (67%). All participants, including two teachers, underwent an initial training on water quality parameters and the citizen science methods that were going to be used, which included theoretical and hands-on experience. During the fieldwork, they were subdivided into groups of four and a water quality kit, as well as a booklet to record the measurements were provided. Next section presents a brief explanation of the calibration, usage and measurement parameters of each method, as well as technical specifications.

During the second month of the project, while conducting the second round of water quality monitoring, interviews were held with the participants. These interviews aimed to gather insights into participants’ perceptions of local water quality and the links to pollution in their environment. This qualitative data helped contextualize the scientific findings within the lived experiences and concerns of the community. After four months of water quality monitoring, participants were invited to a workshop incorporating two main sections. First, water quality data collected over the four months was presented and discussed, providing a platform for participants to reflect on the dynamics of local water quality. Second, experts on Indigenous rights and national environmental law organized discussions on the national legal standards related to Indigenous environmental rights. The workshop facilitated an interactional framing process (Dewulf et al. 2009), enabling participants to interpret the water quality results in the context of national environmental legislation and Indigenous rights.

In the final month, the project’s findings were presented in a public event at the community high school. This event provided an opportunity for community authorities, students, and other stakeholders to discuss the results. Participants shared their impressions, concerns, and suggestions regarding the findings. Observations were documented during the event, and a final round of interviews was conducted with school teachers, students, and community authorities to capture their reflections on the project’s outcomes and potential next steps.

While the study is conceptually informed by a resources nexus perspective, participants were not explicitly prompted to reflect on water–food–energy–ecosystem interdependencies during data collection. Instead, interviews and workshops focused on water quality, environmental change, and Indigenous rights. Nexus linkages were subsequently identified through inductive thematic analysis, based on how participants themselves articulated connections between water quality, food security, health, livelihoods, and cultural practices.

### Water quality monitoring

Sampling followed a purposive and site-specific strategy informed by community knowledge and research objectives. Two monitoring locations were selected collaboratively with participants to capture upstream–downstream contrasts relevant to local exposure and use patterns. Water quality measurements were conducted monthly over a four-month period (March–June 2024), resulting in 46 samples, of which 38 were retained after quality control. Sampling frequency and locations were designed to balance analytical relevance with feasibility within a school-based participatory context.

Water quality assessments included the monitoring of key physicochemical parameters such as temperature, pH, electrical conductivity (EC), total dissolved solids (TDS), salinity and turbidity, complemented by the quantification of nutrients such as nitrate (NO_3_), nitrite (NO_2_) and phosphate (PO_4_) which are often linked to anthropogenic activities in the upstream basin. Sampling sites were selected by student sub-groups based on local relevance and accessibility, georeferenced using the Gaia GPS mobile application, and recorded using standardized field forms.

To ensure accessibility and usability in a community-based context, the project employed low-cost, portable tools: the PC60 Apera Instruments multiparameter probe (Apera-Instruments, n.d.), OASE test strips (OASE, n.d.), sera PO_4_ titration kits (SERA, n.d.) and HydroColor mobile app (Leeuw & Boss, 2018). These instruments were chosen for their affordability, ease of interpretation by non-experts, and previous successful applications in citizen science settings.

The Apera probe was used to measure pH, EC, TDS, salinity, and temperature by immersing the sensor in the water and recording readings once they stabilized. To maintain data reliability, the probe was calibrated each sampling day using manufacturer-provided solutions and protocols, with student teams conducting calibrations under the supervision of trained undergraduate facilitators from the Bolivian Catholic University. OASE test strips were used to estimate NO₂, NO₃, and pH levels. After one minute of submersion, results were compared against a standardized colour chart, with all team members collectively agreeing on interpretation to minimize bias. Phosphate concentrations were determined using Sera titration kits, which involved treating the sample with reagents and comparing the resulting colour to a blank reference using a comparator block. Turbidity and suspended particulate matter (SPM) were estimated using the HydroColor mobile app, which analyses a sequence of images of the sky, a reference grey card, and the water surface. The app processes light reflection and colour data to estimate turbidity, offering a user-friendly, image-based method suitable for participatory fieldwork. Table 1 summarizes the technical specifications and detection ranges of all instruments used.

**Table 1:** Water quality methods technical specifications

Method	Parameter	Range	Resolution	Accuracy
PC60 Apera multiparameter	pH	−2 to 16 pH*	0.01 pH	± 0.01 pH ± 1
	Conductivity	0 to 2000 µS/cm	0.1 µS/cm	± 1%
	TDS	0.1 ppm to 10 ppt	0.01 ppt	± 1%
	Salinity	0 to 10 ppt	0.01 ppt	± 1%
	Temperature	0 to 50˚C	0.1˚C	± 0.5˚C
OASE test strips	pH	6.4 to 8.4 pH	0.4 pH	-
	NO_2_	Presence/absence	-	-
	NO_3_	0 to 250 mg/L	10 mg/L, 150 mg/L**	
Sera test	PO4	0.1 mg/L to 10 mg/L	0.25 mg/L	-
HydroColor app	Turbidity	0 to 80 NTU	1 NTU	± 24%***
	SPM	0 to 80 mg/L	1 mg/L	± 24%****

*The equipment is specified for a pH range of − 2 to 16, allowing measurement of extreme acidic or alkaline solutions beyond the conventional 0–14 scale

**Resolution coarser at higher concentrations, making the test less precise as nitrate increases

***Refers to median percent error in retrieval of turbidity when compared to WISP-3 (Leeuw & Boss, 2018)

****1:1 relationship between turbidity and suspended particulate matter concentrations, thus error similar to that of turbidity

The water quality monitoring phase began on March 21 st, 2024, with an initial training session followed by the first field campaign. Subsequent monthly sampling campaigns were conducted on April 25th, May 29th, and June 24th, resulting in a total of 46 unique water samples. The locations can be found in Fig. 2, where priority was given to sampling areas next to Majawi and Katari River, as water quality in these areas is of high concern for community members.

Field data were initially recorded on paper forms and later digitized upon completion of the monitoring phase. Quality control procedures were applied to exclude any incomplete or inconsistently recorded entries, ensuring that only validated data were included in the analysis. The dataset was then disaggregated by location for descriptive statistical analysis, including measures of central tendency and dispersion (mean, maximum, minimum, 25th and 75th percentiles). A Pearson correlation analysis was also conducted to explore relationships among the monitored parameters. Additionally, results were compared to the national regulatory thresholds established in the *Reglamento en Materia de Contaminación Hídrica* (1995), Bolivia’s primary guideline for water quality in surface water bodies. A temporal analysis was performed to assess whether any observable trends could be linked to the seasonal transition into the dry period over the four-month monitoring period.

### Interviews

The assessment of environmental advocacy draws on 20 semi-structured interviews with participants involved in a citizen science water quality monitoring project in the Indigenous community of Chojasivi, supplemented by workshop notes and observations from the final community event. Three rounds of interviews were conducted on April 25, May 29, and August 28, 2024. Participants were purposively selected (Campbell et al. 2020) based on their active involvement in the project. The sample comprised high school students engaged in water quality data collection (*n* = 12), teachers facilitating the citizen science activities (*n* = 2), and community members who participated in the workshops (*n* = 6). Selection criteria emphasized both the degree of involvement in the initiative and availability during the interview period, ensuring continuity across the project’s core stakeholder groups.

The interviews explored participants’ awareness of water contamination, engagement in citizen science, environmental justice awareness, and community impact. All interviews were transcribed verbatim and subjected to qualitative thematic analysis. To protect participants’ identities, all interviews were pseudonymised by assigning codes to each interview before analysis. Thematic coding was performed to identify key patterns and insights from participants’ narratives. The coding process followed an inductive approach, allowing themes to emerge organically from the data while being guided by a pre-defined framework incorporating the four themes incorporated in the interview’s structure.

The coding framework was structured into four main themes: (1) Perception of water quality and pollution, which assessed participants’ awareness of contamination, observed environmental changes, and perceived impacts on health and livelihoods; (2) Engagement in citizen science, which examined motivations for participation, learning experiences, and hands-on interaction with scientific tools; (3) Environmental justice awareness, focusing on the understanding of environmental rights, attribution of responsibility (community, government, industries), and aspirations for policy change; and (4) Community impact and advocacy, which explored the sharing of knowledge within families, involvement in local initiatives, and barriers to sustained environmental action. Furthermore, an emergent theme, intergenerational environmental injustice was identified, underscoring the long-term implications of environmental degradation affecting the community.

Using NVivo software, transcriptions were systematically coded to facilitate the identification of dominant themes. Each selection was categorized under one or more themes, and a comparative analysis was conducted to examine patterns across different participant groups. The analysis also employed an axial coding approach to explore the relationships between themes, particularly linking citizen science engagement to environmental justice awareness. To enhance the reliability of the analysis, intercoder reliability was ensured by involving two researchers in the coding process. Discrepancies were discussed and resolved through consensus. Member-checking was also conducted by sharing preliminary findings with a subset of participants to validate interpretations. Additionally, reflexive journaling (Meyer & Willis, 2019) was maintained throughout the analysis to document researcher biases and ensure transparency.

The final step of the data analysis involved integrating quantitative and qualitative findings to provide a comprehensive understanding of the project’s outcomes. The water quality data was contextualized with the community’s perceptions and concerns, revealing how water quality scientific evidence influenced local environmental justice awareness. This analysis also helped to identify how the community’s engagement with the data could inform future actions or public participation in environmental policymaking. Ethical approval for this study was obtained from the Bolivian Catholic University review board. Free Prior and Informed Consent (FPIC) was obtained from all participants before data was collected. Confidentiality was assured by pseudonymising all participant responses and through the secure storage of data.

This study does not seek statistical generalization beyond the case of Chojasivi. Instead, it adopts an analytical generalization approach, whereby insights derived from this empirically grounded case are used to refine and contribute to broader theoretical discussions on citizen science, Indigenous environmental justice, and participatory water governance. The findings are thus transferable at the level of concepts and mechanisms rather than populations, offering relevance for similarly structured contexts characterized by Indigenous marginalization, environmental contamination, and weak institutional accountability.

## Results

The Results section presents empirical observations and participant narratives; their theoretical, justice-oriented, and implications are examined in the Discussion section.

### Water quality results

A total of 46 water samples were collected through four monthly campaigns; of these, 38 (83%) passed quality control checks and were included in the final analysis. The most common exclusion reason was inaccurate geolocation, likely due to mobile app limitations. Sampling focused on areas near the Majawi and Katari rivers, which were identified by community members as high concern zones (see Fig. 2). Although a monitoring period of at least one year would have been preferable to understand the dynamics of water quality, this project had a short duration due to project funding supporting the transportation of the students to the sampling sites and the school calendar. Descriptive statistics for each parameter are presented in Table 2. Values exceeding Bolivian regulatory thresholds for Class C surface waters (Reglamento en Materia de Contaminación Hídrica, 1995) are indicated in bold. Total dissolved solids (TDS), phosphate (PO₄), and turbidity showed the most frequent exceedances of water quality thresholds, with the highest concentrations recorded in the Katari River Basin.

**Table 2:** Summary of water quality parameters by sampling site and campaign

		Majawi						Katari River					
Parameter	Month	Min	Max	Median	25th P	75th P	Count	Min	Max	Median	25th P	75th P	Count
Temperature [°C] (PC60 Apera Instruments)	MAR	23	24.2	23.7	23.4	23.9	6	17.4	19.7	18.2	17.5	19.5	5
	APR	17.1	17.9	17.5	17.3	17.7	2	17.3	20.5	19.1	18.2	19.8	3
	MAY	11.6	13.6	12.6	12.1	13.1	2	8.4	12.8	10.5	9	12.1	4
	JUN	8.5	14.4	12	11.5	12.8	5	8.3	9.8	8.6	8.4	9.3	5
pH (PC60 Apera Instruments)	MAR	8.2	8.5	8.3	8.2	8.5	6	7.5	7.9	7.6	7.5	7.6	5
	APR	7.7	7.7	7.7	7.7	7.7	3	8	8	8	8	8	3
	MAY	**9**	**9.1**	**9.1**	**9**	**9.1**	3	8.2	8.4	8.3	8.2	8.4	4
	JUN	8.8	**10.4**	**9.5**	**9**	**10.3**	5	8.1	8.6	8.4	8.3	8.6	5
Conductivity [µS/cm] (PC60 Apera Instruments)	MAR	**620**	**707**	**654**	**633**	**679.5**	6	**528**	**667**	**637**	**619**	**654**	5
	APR	**1068**	**1068**	**1068**	**1068**	**1068**	1	**1180**	**1384**	**1264**	**1222**	**1324**	3
	MAY	**749**	**969**	**864**	**806.5**	**916.5**	3	**1498**	**1700**	**1621**	**1572.3**	**1658.8**	4
	JUN	**960**	**1690**	**1257**	**1015**	**1330**	5	**1700**	**1959**	**1870**	**1811.8**	**1908**	4
TDS [ppt] (PC60 Apera Instruments)	MAR	443	**501**	464.5	450.8	473.8	6	371	494	443	429	457	5
	APR	**728**	**932**	**830**	**779**	**881**	2	**820**	**1040**	**919**	**869.5**	**979.5**	3
	MAY	**533**	**684**	**609**	**571**	**646.5**	3	**1130**	**1190**	**1155**	**1137.5**	**1175**	4
	JUN	**684**	**995**	**892**	**780**	**929**	5	**1170**	**1890**	**1360**	**1300**	**1390**	5
Salinity [ppt] (PC60 Apera Instruments)	MAR	0.3	0.4	0.3	0.3	0.3	6	0.3	0.4	0.3	0.3	0.3	5
	APR	0.5	0.8	0.7	0.6	0.7	3	0.6	0.7	0.7	0.6	0.7	3
	MAY	0.4	0.5	0.4	0.4	0.5	3	0.8	0.8	0.8	0.8	0.8	4
	JUN	0.5	0.7	0.6	0.6	0.6	5	0.4	1	0.9	0.8	1.0	5
PO_4_ [mg/L] (Sera)	MAR	**0.5**	**1.5**	**0.8**	**0.5**	**1.2**	6	0.1	**1.3**	0.1	0.1	**0.5**	5
	APR	**1**	**2**	**2**	**1.5**	**2**	3	**2**	**4**	**4**	**3**	**4**	3
	MAY	0.3	**0.5**	0.3	0.3	0.4	3	**2**	**2**	**2**	**2**	**2**	4
	JUN	0.1	**1**	0.1	0.1	0.3	5	0.3	**2**	**0.5**	**0.5**	**0.5**	5
NO_3_ [mg/L] (OASE)	MAR	0	**10**	0	0	0.0	6	0	**10**	0	0	0	5
	APR	0	**10**	**10**	5.0	**10**	3	0	**10**	0	0	5	3
	MAY	0	**10**	**10**	5.0	**10**	3	0	**25**	**10**	7.5	13.8	4
	JUN	0	0	0	0	0	5	0	0	0	0	0	5
pH (OASE)	MAR	6.5	7.6	6.8	6.8	7.1	6	6.8	7.2	7.2	6.8	7.2	5
	APR	8	9.6	8.8	8.4	**9.2**	2	7	8	7.5	7.3	7.8	2
	MAY	6.4	7.6	7.2	6.8	7.4	3	7	8.4	7.8	7.5	8.1	4
	JUN	6.8	7.2	7.2	7	7.2	5	6.8	8	7.6	7.2	7.6	5

The HydroColor mobile app proved unsuitable for this context, as turbidity levels frequently exceeded their 80 NTU detection limit. Independent measurements taken with the Hanna HI9829-13042 multiparameter recorded turbidity values averaging 240 NTU and reaching over 1000 NTU at critical points, far exceeding the legal maximum of 50 NTU. These values indicate persistent and severe turbidity issues, which community elders described as a recent development compared to past conditions.

To contrast the results with national legislation, the concentrations obtained were compared with the *Bolivian Reglamento en Materia de Contaminación Hídrica*, specifically the values for Class C which indicates that their use is for animal consumption.

**Fig. 4:**
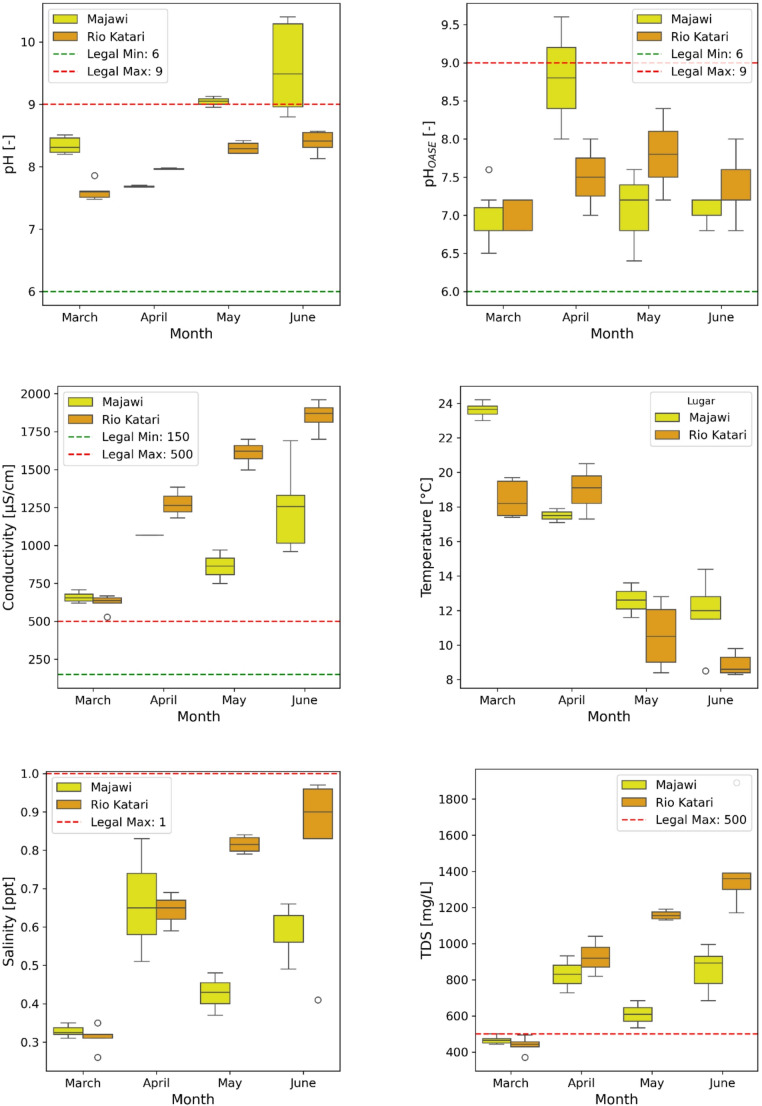
Box plots for each monitored parameter, divided by sampling site and date

**Fig. 5:**
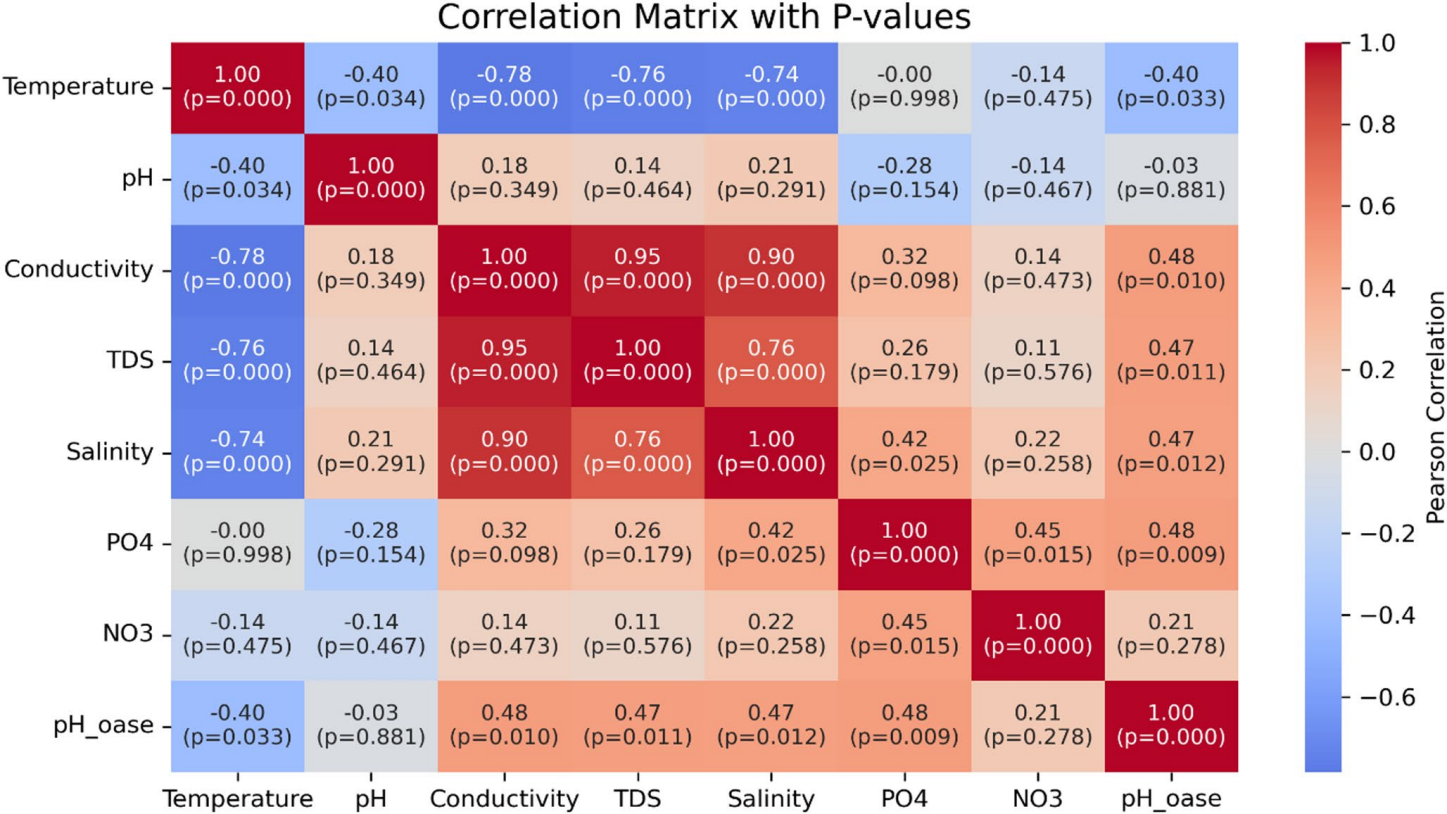
Pearson correlations and p-values between monitored water quality parameters

Conductivity, TDS, and salinity showed a clear temporal trend: lowest values were recorded in March during the rainy season, followed by steady increases through June, coinciding with the onset of the dry season (Fig. 4). This pattern reflects reduced dilution and higher pollutant concentrations due to declining river flows. The trend is supported by strong negative correlations between temperature and conductivity (*r* = − 0.78), TDS (–0.76), and salinity (–0.74), all statistically significant (*p* < 0.001), as shown in Fig. 5.

Nutrient levels also reflected seasonal dynamics. A sharp PO₄ spike in April (up to 4.0 mg/L) in Katari River likely resulted from first-flush runoff events following the rainy season. Similarly, NO₃ levels peaked in May, potentially due to delayed input from agricultural and urban sources. These episodic elevations suggest increased nutrient loading during transitional hydrological periods, underscoring the risk of eutrophication in downstream ecosystems such as Lake Titicaca.

While NO₃ concentrations remained mostly within the national limit of 10 mg/L, PO₄ consistently exceeded the 0.5 mg/L threshold, especially in Katari River. TDS levels also surpassed the 500 mg/L standard across multiple sites and time points. NO₂ was detected in nine samples (24%), primarily during March and April, suggesting the presence of fresh organic pollution early in the monitoring period.

The pH values recorded by the Apera probe remained within acceptable limits but showed a gradual increase toward alkaline conditions in June, particularly in Katari River. This trend likely reflects reduced water volume and concentration of alkaline substances during the dry season. In contrast, pH values from the OASE test strips were inconsistent, showing significant variability even when probe readings were stable. Calibration checks with the Hanna multiparameter in March (range 7.5–8.8) confirmed the higher reliability of the Apera readings.

Overall, the data collected by citizen scientists demonstrated reasonable internal consistency. Parameters like EC, TDS, and salinity exhibited tight interquartile ranges, especially in Katari River, indicating reproducibility. In contrast, greater variability in PO₄ and NO₃, particularly in Majawi, may reflect localized pollution events or limitations in strip-based testing. Despite these limitations, the dataset captured clear spatial and temporal trends consistent with regional hydrological dynamics and community-reported concerns. These findings document repeated exceedances of national water quality thresholds in downstream locations used by Indigenous communities.

### Insights from indigenous citizen scientists

Thematic analysis of 20 semi-structured interviews is presented through four core themes that reflect how participants engaged with water quality issues through the citizen science for environmental justice advocacy initiative: (1) perceptions of water quality and pollution, (2) engagement in citizen science, (3) environmental justice awareness, and (4) community impact and advocacy. In addition, the analysis identifies an emergent theme of (5) intergenerational environmental injustice, highlighting how long-term degradation has disrupted ecological memory and transferred environmental burdens across generations. A final section offers a resource nexus reflection, examining how participants implicitly connected water quality to food security, ecosystem health, and broader structural interdependencies, a summary results is presented in Table 3.

#### Perceptions of water quality and pollution

All participants (20/20) expressed strong concerns about the water contamination levels and their consequences, describing both visible and experiential changes in their environment. This was grounded in direct sensory observation, such as dark colour, unpleasant odour, and visible waste, and was reinforced by lived experiences of ecological decline. One participant remarked, “Now it’s very polluted. Before we could use that water, now not even animals can drink it” (280824_GJ2_JEGF - DAL). Several others recalled witnessing floating debris and foam, with one noting, “When we entered the lake, we saw floating trash and white foam” (250424_CC6).

This recognition of deterioration extended to health concerns and the disappearance of aquatic life. 12 participants linked declining water quality with gastrointestinal illnesses or the loss of fish, indicating an understanding of the connection between environmental harm and community well-being. One of the interviewees recalled, “Our grandparents fished in these rivers, but now there’s nothing left due to pollution” (290524_CS2), emphasizing the tangible loss of ecosystems. Moreover, 40% of interviewees observed fluctuations in pollution levels tied to weather patterns, suggesting a nuanced grasp of pollution as a dynamic and temporally variable phenomenon, one respondent noted, “Each test showed different contamination levels depending on the weather, which was interesting” (290524_CS3). These insights reflect participants’ lived experiences of environmental degradation and unequal exposure to pollution.

#### Engagement in citizen science

Nineteen of the twenty Participants described their engagement in citizen science as personally meaningful, particularly in terms of learning, confidence, and legitimacy in discussing environmental issues. Learning to use water quality instruments was repeatedly described as empowering. One participant stated, “Using the water testing kits made me feel like a real scientist, and now I understand how to measure contamination levels” (290524_CS1). while another noted, “I never thought we could do these kinds of studies like scientists do” (290524_CS5). Beyond technical skill acquisition, participants expressed a growing sense of agency and legitimacy in contributing to environmental knowledge. The act of comparing contamination levels across sampling sites led many to interpret pollution as a complex and spatially variable issue, insights that extended beyond conventional environmental education.

However, 6 participants also identified challenges to sustained engagement, including the lack of follow-up, limited access to equipment, and uncertainty about how to continue monitoring. As one noted, “It was exciting at first, but without proper guidance, it’s hard to know what to do next” (280824_GJ1_LSC). This tension stresses the limitations of short-term participatory initiatives when not embedded within broader institutional frameworks.

Beyond technical skills, engagement in the project reinforced participants’ confidence in communicating environmental concerns. One participant explained, “Seeing the data we collected gave me confidence to speak about water pollution in our community meetings” (290524_CS7).

These experiences point to strengthened procedural participation and recognitional processes associated with environmental justice.

#### Environmental justice awareness

Thirteen participants articulated perceptions of unfair exposure to pollution, lack of consultation, and limited institutional response. While few used legal terminology, many identified pollution as structurally unfair and demanded accountability. There was a clear recognition of corporate and governmental responsibility in environmental matters. One participant noted, “City waste reaches us and affects us, but no one asks our opinion” (280824_GJ1_LSC - NAA). Another participant stated, “companies polluting our water should be held accountable through stricter laws” (280824_GJ2_JEGF).

This sense of injustice was not limited to moral outrage, it reflected an emergent awareness of spatial and political exclusion. Participants described a lack of consultation, limited enforcement of environmental laws, and unequal exposure to environmental risks. Notably, only 4 participants expressed familiarity with formal environmental rights or legal instruments, indicating a disconnect between local perceptions of injustice and institutional avenues for redress. For instance, one interviewee admitted, “I think everyone has the right to clean water, but I don’t know how to enforce it” (290524_CS2). While participants frequently described pollution as unfair and expressed frustration with institutional inaction, few demonstrated familiarity with formal environmental rights or legal mechanisms for redress. These findings highlight a gap between locally generated environmental concerns and knowledge of formal legal frameworks.

#### Community impact and advocacy

Fifteen participants described sharing monitoring results informally within their families, schools, and community meetings. Several reported sharing monitoring results at home, leading to increased caution regarding water use. One participant explained, “After learning, I explained to my mom why the water was bad and shouldn’t be drunk” (290524_CS1). Such exchanges point to a “spillover effect,” where knowledge produced in participatory spaces diffuses through informal channels.

In addition to interpersonal dialogue, 9 participants referenced collective initiatives inspired by the project. These included river clean-up efforts, classroom-based campaigns, and local awareness activities. For example, “With my group we talked about launching a campaign to clean and educate the community” (250424_CC8). However, enthusiasm was often tempered by the perceived absence of leadership and institutional support. As one participant lamented, “We inform the authorities, but nothing changes” (290524_CS1).

These findings reveal both the promise and fragility of citizen-led environmental awareness and informal advocacy efforts: while the initiative fostered grassroots momentum, structural barriers may constrain its long-term impact. These accounts show that knowledge and concern generated through the project circulated primarily through informal community channels rather than formal governance structures.

#### Intergenerational environmental injustice

Eight participants explicitly reflected on intergenerational environmental change, emphasizing how the deterioration of water quality has disrupted the continuity between past and present ecological conditions. Participation in the citizen science initiative prompted them to engage in conversations with parents and grandparents about the historical state of the river. As one participant recalled: “My grandparents told me there used to be many fish and you could swim. Now that’s no longer possible” (250424_CS345).

This theme was marked by a perceived rupture, namely the loss of ecological memory and the normalization of environmental degradation among younger generations. The statement, “Young people don’t know how it used to be” (280824_GJ1_LSC - NAA), illustrates how environmental decline has affected not only material conditions but also shaped cultural identity and expectations.

Participants also expressed a sense of inherited responsibility for environmental problems they did not cause. One remarked, “Pollution started years ago, but now we’re the ones dealing with the consequences” (290524_CS5). Several linked this shift to the erosion of traditional water stewardship values, particularly those embedded in Indigenous worldviews: “In our culture, it was forbidden to contaminate water, but now people don’t respect that” (280824_GJ1_LSC - NAA).

Participants described environmental change across generations, emphasizing the loss of ecological memory and the transfer of environmental burdens to younger community members.

**Table 3:** Findings from participant interviews on water quality and environmental justice

Theme	Description	Frequency (*n* = 20)
Perceptions of Water Quality and Pollution	Recognition of visible degradation and its effects on health, ecology, and daily life.	20/20
Engagement in Citizen Science	Hands-on learning increased environmental awareness and perceived scientific legitimacy.	19/20
Environmental Justice Awareness	Participants identified unequal pollution burdens and political exclusion.	13/20
Community Impact and Advocacy	The initiative inspired dialogue, behaviour change, and modest collective action.	15/20
Intergenerational Environmental Injustice	Loss of ecological memory and cultural reference points due to long-term degradation.	8/20

#### Resource nexus awareness and interconnected impacts

Although participants were not explicitly asked about the interdependencies among water, food, and energy systems, 12 of the 18 interviews revealed an implicit understanding of these linkages. Most references emerged through accounts of how water pollution has disrupted food security, particularly through the decline of fisheries, reduced crop yields, and negative impacts on livestock. As one participant recalled, “We used to catch fish for our meals, but now there’s nothing in the river.” (280824_GJ2_JEGF – DAL). Another described the deteriorating quality of irrigation water and its effect on agricultural productivity: “We can’t grow like before because the water burns the plants.” (250424_CS345). Additionally, 8 participants linked poor water quality to gastrointestinal illnesses, further highlighting the interconnected consequences of environmental degradation. In these narratives, the loss of aquatic biodiversity was framed not only as an ecological issue but also as a driver of food and economic insecurity.

A smaller group of participants (20%) referenced structural contributors to pollution, such as mining runoff and untreated discharges from upstream urban centres. These reflections, although less frequent, demonstrated an emerging awareness of how upstream industrial and urban activities shape downstream ecological and social vulnerabilities. In several cases, participants framed these dynamics in terms of injustice, pointing to asymmetries in exposure, agency, and accountability.

Taken together, participants’ narratives reveal interconnected impacts of water quality degradation on health, food production, and livelihoods.

## Discussion

This section focuses on three key findings emerging from the empirical results: (i) persistent distributive water pollution injustices affecting downstream Indigenous communities; (ii) the role of citizen science in strengthening procedural and recognitional justice processes without producing institutional or distributive change; and (iii) the emergence of intergenerational and nexus-related dimensions of environmental injustice. Rather than reiterating descriptive results, this section interprets their implications for Indigenous environmental justice and participatory water governance.

### Citizen science insights on current water quality conditions in the Katari river basin

Persistent exceedances of national water quality standards across monitoring sites indicate systemic pollution rather than episodic contamination. Spatial contrasts between upstream and downstream locations reflect structural exposure patterns shaped by mining, urban wastewater, and industrial discharge. From an environmental justice perspective, these findings document a clear distributive injustice, whereby downstream Indigenous communities bear disproportionate environmental burdens generated outside their territories. Repeated exceedances of thresholds for turbidity, total dissolved solids, and nutrients constitute empirical evidence of unequal exposure rather than isolated environmental incidents. These conditions pose direct risks to ecosystem integrity, food systems, and public health, particularly in Lake Titicaca. The findings underscore the need for institutionalized, long-term monitoring and enforcement mechanisms capable of addressing cumulative pollution dynamics rather than short-term fluctuations.

### Citizen science through the lens of indigenous environmental justice

Interpreting the findings through a multidimensional environmental justice framework clarifies both the contributions and the limitations of the citizen science initiative. The project generated evidence relevant to distributive injustice, strengthened procedural and recognitional justice processes, and revealed intergenerational dimensions of harm, while leaving broader institutional justice outcomes largely unaddressed.

#### Citizen science as situated knowledge production

Participants from the Indigenous community of Chojasivi did not merely collect data; they interpreted pollution trends through the lens of daily life, cultural memory, and ecological observation. This situated environmental knowledge extended beyond anecdotal concern, with participants identifying seasonal pollution patterns, spatial variability, and ecosystem decline. These findings align with existing research showing that citizen science enhances environmental literacy when grounded in local realities (Bonney et al. 2009; Pandya 2012). The data demonstrate how citizen science can amplify community voices and challenge knowledge hierarchies at the community level, while remaining insufficient to alter institutional hierarchies of expertise or generate enforceable environmental justice outcomes. Yet, scientific participation alone does not guarantee influence. Several participants voiced frustration over the lack of official response to their water quality data and concerns. This mirrors findings from other contexts where citizen-generated knowledge remains undervalued in formal decision-making (Ottinger 2010). The findings indicate that citizen science primarily strengthened procedural and recognitional dimensions of environmental justice, while leaving the structural conditions generating pollution unchanged. Bridging this gap requires institutional mechanisms that validate and integrate community knowledge into environmental policy frameworks, ensuring that local perspectives inform decisions.

#### Environmental justice and legal empowerment

Although many participants recognized the injustice of bearing the burden of upstream pollution, few were aware of formal environmental rights or legal mechanisms for accountability. These findings reflect broader challenges faced by Indigenous communities in Latin America, where environmental harms often intersect with political marginalization and limited access to legal resources (Schlosberg et al., 2010). The gap between awareness and legal literacy limits communities’ capacity to pursue redress. To address this, citizen science programs exploring environmental justice must integrate environmental legal education. Programs that integrate scientific monitoring with legal training have proven effective elsewhere, enabling communities to leverage data for environmental governance and, when necessary, litigation (Ottinger 2010). While citizen science can initiate engagement, long-term environmental justice advocacy necessitates sustained capacity-building and institutional support. At the same time, structured community organisations and leadership development are essential for maintaining momentum and ensuring that environmental monitoring translates into enduring policy impact (Israel et al. 2019).

Participants must not only collect data but also to understand how to strategically use it to demand action, engage institutions, and, when necessary, pursue legal remedies. This is particularly relevant in Bolivia, where the Constitution recognizes Indigenous territorial and water rights, yet institutional mechanisms for enforcement remain weak and unevenly applied. This distinction reinforces the importance of treating environmental justice not as an automatic outcome of participation, but as a multidimensional process shaped by institutional power, legal accessibility, and governance responsiveness.

While the participant’s perceptions reflect a growing awareness of environmental injustice, they did not consistently translate into knowledge of formal legal rights or access to institutional mechanisms for redress, highlighting a gap between justice awareness and justice realization. These findings indicate that the inclusion of environmental rights–related information in citizen science programs could support community participation, by situating locally generated data within existing legal and governance contexts.

Overall, the findings caution against equating participatory monitoring with environmental justice outcomes. While citizen science can strengthen awareness, recognition, and procedural engagement, distributive injustices and institutional accountability remain structurally constrained. Recognizing this distinction is essential to avoid normative overreach and to situate citizen science within broader struggles for environmental justice rather than as a standalone solution.

#### Intergenerational justice and ecological memory

Beyond its empirical relevance, the identification of intergenerational environmental injustice in this study constitutes a theoretical contribution to environmental justice scholarship by foregrounding ecological memory and cultural continuity as central dimensions of justice across time.

The theme of intergenerational environmental injustice was noticeable across interviews. Participants described a rupture in ecological memory, the disconnection between past ecological conditions and current realities. Based on discussion about pollution triggered through this citizen science project, elders recalled clean rivers and abundant fish, while youth grew up with contamination as the norm. This loss of reference points not only reflects environmental degradation but also reshapes cultural identity. These narratives speak directly to an intergenerational dimension of environmental injustice, in which environmental burdens generated by past and ongoing pollution are transferred to younger generations, alongside the erosion of ecological memory and cultural reference points. Citizen science emerged as a space for intergenerational dialogue, allowing younger participants to engage with elders and reconstruct a collective memory of the environment. These findings echo with scholarship on environmental and social–ecological memory, which emphasizes the role of intergenerational learning in sustaining cultural continuity, resilience, and stewardship in socio-ecological systems (Barthel et al. 2010). In this sense, citizen science functioned less as a justice-delivery mechanism than as a catalyst for intergenerational recognition and dialogue, enabling communities to reconstruct ecological baselines and articulate long-term environmental harm as an injustice spanning generations.

#### Nexus-informed justice

Participants’ narratives reveal an implicitly articulated resource nexus, in which water quality degradation was linked to food security, health, livelihoods, and cultural practices. These linkages are interpreted here as governance-relevant implications rather than independent empirical findings. Participants’ reflections revealed an implicit understanding of the resource nexus: water pollution was repeatedly linked to food insecurity, health risks, and livelihood impacts. While participants did not articulate this in formal systems language, their narratives illustrated how environmental degradation cascades across domains. These insights emphasize the importance of adopting a nexus approach in water governance, especially in contexts where multiple vulnerabilities converge. From a policy perspective, these findings suggest that citizen science represents an opportunity to reflect on the interconnections of Sustainable Development Goals (Gharesifard et al. 2025). Pollution in the Katari Basin undermines SDG 6 (Clean Water), SDG 2 (Food Security), SDG 3 (Health), SDG 13 (Climate Action), and SDGs 14/15 (Biodiversity). Yet, without institutional responsiveness (SDG 16), these interconnections remain unaddressed. Environmental monitoring must be accompanied by political accountability to translate data into justice. In addition, acknowledging and integrating local knowledge and lived experiences enables policymakers and practitioners to design more context-sensitive and just interventions that address multiple vulnerabilities simultaneously.

## Conclusions

This study investigated the implementation of a citizen science initiative in the Katari River Basin, focusing on local water quality monitoring and how participants interpreted the findings in relation to environmental justice and Indigenous rights. While the water quality monitoring covered only four months, which is insufficient to draw long-term conclusions about seasonal or annual trends, it represents a critical first step in generating empirical data in a region where formal water quality monitoring remains sparse and inequitable. Accordingly, the water quality results are interpreted as indicative of persistent contamination pressures rather than as representative of long-term temporal dynamics, and no claims are made regarding annual or interannual trends. Despite this limitation, the findings revealed that water quality thresholds for phosphates, total dissolved solids, and electrical conductivity frequently exceeded national standards, signalling systemic environmental risks for downstream Indigenous communities.

Furthermore, the study shows that participants, through direct engagement in sampling and contextualizing the results against national standards, developed locally grounded understandings of pollution patterns and voiced concerns about their social, ecological, and health implications. The initiative fostered environmental awareness, intergenerational dialogue, and the recovery of ecological memory. However, its capacity to advance environmental justice remains constrained by structural limitations. Key barriers included limited legal literacy, limited institutional responsiveness, and the absence of formal mechanisms for accountability. Participants expressed frustration with the lack of state engagement and the inability to translate scientific evidence into concrete action.

Drawing on a resource nexus lens, the findings also highlight the interdependence of water quality, food systems, and cultural integrity, emphasising the need for rights-based, community-anchored approaches to environmental governance in Indigenous contexts.

This research contributes to ongoing debates on the role of citizen science in environmentally and politically contested contexts. It highlights the potential of citizen science to produce credible data and stimulate critical reflection, while also exposing the systemic constraints that limit its transformative potential. Future research should focus on linking community-generated data to legal and policy frameworks, strengthening legal literacy, and advancing long-term partnerships among communities, civil society, and institutions to enhance the impact of participatory environmental monitoring in the pursuit of environmental justice. While the findings are grounded in a single case study and a limited monitoring period, they offer analytically generalizable insights into how citizen science shapes environmental justice processes under conditions of structural constraint.
